# Examining Age as Moderating Associations Between Methamphetamine Abstinence and Cognitive Function in People with HIV

**DOI:** 10.3390/v18090953

**Published:** 2026-08-31

**Authors:** Victoria O. Chentsova, Jennifer E. Iudicello, Anya Umlauf, Akashi K. Suon, Igor Grant, Mariana Cherner

**Affiliations:** 1 San Diego State University/University of California San Diego Joint Doctoral Program in Clinical Psychology, San Diego, CA 92120, USA; 2 HIV Neurobehavioral Research Program, University of California San Diego, San Diego, CA 92093, USA; jiudicello@health.ucsd.edu (J.E.I.); aumlauf@health.ucsd.edu (A.U.); igrant@health.ucsd.edu (I.G.); mcherner@health.ucsd.edu (M.C.); 3 Department of Psychiatry, University of California San Diego, San Diego, CA 92093, USA; 4 Department of Psychology California State University, Dominguez Hills, CA 90747, USA; asuon3@toromail.csudh.edu

**Keywords:** HIV, neurocognition, methamphetamine use disorder, recovery

## Abstract

Objective: This study examined associations of days of methamphetamine (METH) abstinence with neurocognitive functioning and whether age moderates this relationship in people with HIV (PWH). Methods: Retrospective data from 122 PWH with lifetime METH use disorder (MUD) were analyzed. Linear, piecewise, and curvilinear models were compared to characterize the association between length of METH abstinence and Global neuropsychological test T-scores. An interaction term between METH Abstinence Days and Age Group (<42, 42–48, >49) was added to the best model to test moderation both globally and by domain. Results: A curvilinear model with log-transformed METH Abstinence Days provided the best model fit, with higher METH Abstinence Days associated with higher Global T-scores and a decreasing positive slope over time. Age Group did not moderate Global T-scores. At the domain level, Age Group moderated associations between METH Abstinence Days and Information Processing Speed, with steepest slopes in the youngest age group. Conclusions: Length of METH abstinence is curvilinearly associated with neurocognitive functioning, with the steepest associations occurring in early abstinence, and is largely independent of age. These results emphasize that most neurocognitive recovery in domains impacted by MUD occurs during early abstinence across age groups and may inform the timing of neurocognitive assessment and intervention.

## 1. Introduction

Breakthroughs in modern medicine, and particularly the advent of antiretroviral therapies (ART), have transformed HIV from a terminal diagnosis to a manageable chronic condition. Despite the transformative success of ART in suppressing HIV and extending life expectancy, neurocognitive complications associated with HIV persist [1], with some studies suggesting that neurocognitive impairment (NCI) may affect up to 50% of people with HIV (PWH) globally [2]. The real-world consequences of even milder forms of NCI, including challenges in medication adherence in addition to financial management, employment, driving, and other activities of daily living, are well established and may limit independence in PWH [3,4,5,6]. Among factors contributing to these challenges is substance use, including methamphetamine (METH) use.

METH use is disproportionately prevalent among people with HIV (PWH), with some studies suggesting METH use is three to four times more prevalent compared to the general population [7]. The use of METH among PWH is associated with multiple barriers to management of HIV care, including posing challenges to ART adherence, medication for addiction treatment (MAT) adherence, and medical appointment engagement, collectively leading to higher risk of HIV-related health complications and poorer mental health outcomes [8,9,10]. Among the primary mechanisms contributing to these negative consequences are the potent neurotoxic effects of METH on the central nervous system resulting from dysregulation in dopaminergic and serotonergic systems and subsequent neurocognitive challenges [11,12]. Studies thus far have provided substantial evidence of worse neurocognitive outcomes in the context of comorbid HIV and METH use [13,14]. Thus, understanding trajectories of neurocognitive recovery with METH abstinence is of considerable clinical and public health importance, particularly among PWH, who are already vulnerable to brain dysfunction.

Evidence from meta-analyses in the general population shows that METH is associated with pronounced deficits in impulsivity/reward processing and social cognition, moderate impairment across attention, executive functioning, verbal fluency, verbal learning and memory, and relative sparing of visuospatial abilities [15]. Research demonstrates that extended abstinence from METH is associated with at least partial recovery of neurobiological and subsequent cognitive functioning [16]. Neuroimaging studies provide evidence for recovery on a biological level, with various implicated mechanisms, from stronger network connectivity in parietal and executive control systems, to greater glucose metabolism and higher dopamine transporter availability, to associations between higher gray matter volume, namely in the left cerebellum, and more days of abstinence [17,18,19,20]. Behaviorally, longer abstinence is associated with recovery, particularly in frontally mediated abilities, including executive functions such as planning and set shifting, as well as attentional and inhibitory control [21,22]. At the same time, there is evidence of long-term neurobiological changes and cognitive dysfunction sustained after a year or more of continuous abstinence, suggesting that certain effects of chronic METH exposure may be long-lasting [23]. Overall, studies across substance classes generally suggest that neurocognitive recovery tends to be time-dependent and front-loaded, consistent with a curvilinear model of recovery [24,25,26].

Importantly, much of this literature was generated in samples of people without HIV, leaving open the questions of how these associations show up in PWH. For PWH, the recovery following METH abstinence may be substantially altered or associated with distinct outcomes. The neuroimmune burden of HIV infection, from chronic neuroinflammation and ongoing CNS viral reservoirs to legacy effects of neuropathologic changes developed prior to treatment, may impose a ceiling on recoverable function and slow the pace of improvement [27,28]. Cross-sectional studies have demonstrated an additive burden on neurocognition in individuals with HIV who use METH as compared to either condition alone [13,29].

Further complicating trajectories of recovery in PWH is aging. In general, rates of substance use, including METH use, decline among older populations [30,31]. However, recent evidence points to rising trends among middle-aged and older adults in both treatment admissions for METH use as well as METH-related overdose mortality [7,32]. This trend may reflect the impact of improvements in healthcare, enabling PWH who use METH to survive into older age [1]. Nevertheless, aging and HIV are each independently associated with neurocognitive vulnerability, and a substantial literature suggests their effects converge, such that older PWH exhibit disproportionately poorer neurocognitive and everyday functioning outcomes than would be expected from either factor alone [27,33]. METH adds a third insult that targets many of the same mechanisms implicated in both HIV and normal aging [34]. Beyond its impact on baseline cognitive function, age may also shape the capacity for recovery itself. Preliminary evidence in METH-using PWH suggests that the benefits of abstinence may be age-dependent, with longer abstinence associated with better neurobehavioral performance across frontal systems (i.e., disinhibition, executive function) in younger but not older adults [35]. Relatedly, a remote history of METH dependence has been found to exert a detrimental “legacy” effect on neurocognition (e.g., memory) and real-world functioning among older but not younger PWH, even years after use has ceased [34]. Taken together, these findings raise the hypothesis that recovery trajectories following METH abstinence may be steeper and more complete in younger PWH, and flatter or more constrained in older PWH for whom the cumulative burden of aging, HIV, and prior METH exposure may limit the degree of attainable neurocognitive restoration.

The present study, thus, seeks to address these gaps by examining associations between METH abstinence and neurocognitive functioning, as well as the moderating role of age in people with HIV. We first aimed to examine trajectories of recovery, exploring linear, piecewise, and logarithmic associations between abstinence from METH and neurocognitive test performance. In line with research on neurocognitive recovery more broadly, we expected a curvilinear association, with rate of recovery slowing over time, to best represent the association between abstinence from METH and neurocognitive test performance [36,37]. Following identification of the best-fitting model, model parameters were examined on a global level. Research and clinical best practice thus far have largely focused on the first six months to year of abstinence as the time of greatest expected recovery [24,38]. Finally, we examined whether age moderates the association between abstinence from METH and neurocognitive test performance. Given synergistic effects of aging and HIV on neurocognitive functioning, and prior evidence suggesting that age may attenuate abstinence-related gains in METH-using PWH, we anticipated that recovery trajectories would be steeper in younger individuals [34].

## 2. Methods

### 2.1. Participants and Procedures

We analyzed retrospective data from 122 people with HIV (PWH) collected across HIV Neurobehavioral Research Program (HNRP) studies between 2004 and 2020. Inclusion required a lifetime history of METH use disorder (MUD) according to the *Diagnostic and Statistical Manual of Mental Disorders* (DSM) IV or V, and reported METH use within two years of the study visit. All participants had comprehensive neuromedical, neurocognitive, substance use, and psychiatric data. See Table 1 for a summary of all the variables included in this study.

To maintain study generalizability, exclusion criteria were minimal and primarily defined by parent studies [1,10,39,40]. Parent studies consistently excluded participants for the presence of any known major neurological or psychiatric conditions that could confound neurocognitive performance (e.g., seizure disorders, schizophrenia). Cases were excluded if neurocognitive testing was not completed in English, or if participants met criteria for a current or past 12-month substance use disorder diagnosis for other drugs, except cannabis use disorder (CUD). Exceptions were made for cannabis given the high rates of use in PWH, and effects were examined and controlled for where necessary.

Demographic information was obtained via a standardized self-report questionnaire, including questions regarding age, years of education, sex, and race and ethnicity. Participants were further segmented into three age groups, reflecting tertiles of the sample distribution: <42, 42–48, and 49+.

### 2.2. Demographics and Clinical Characteristics

#### 2.2.1. HIV Characteristics

HIV serostatus was confirmed using enzyme-linked immunosorbent assays with Western blot confirmation. HIV characteristics and other medical history were collected using a clinician-administered neuromedical questionnaire and included information on antiretroviral therapy (ART) usage as well as historical immunological function as represented by reports of lowest-ever (nadir) CD4 counts. Routine in-house clinical chemistry panels, complete blood counts, rapid plasma reagin, and CD4+ T cell count (flow cytometry), performed in certified clinical laboratories on collected blood specimens, were used to represent current immunological function and viral suppression. HIV viral load was dichotomized as detectable vs. undetectable at a lower limit of quantitation (LLQ) of 50 copies/mL.

#### 2.2.2. Neurocognitive Functioning

Neurocognitive T-Scores were calculated from a validated battery of tests assessing neurocognitive function across seven domains: Verbal Fluency, Executive Function, Processing Speed, Learning, Delayed Recall, Working Memory/Attention, and Motor Skills. Individual test scores were transformed into normally distributed T-scores, adjusted for age, education, sex, and race/ethnicity, and then averaged into domain T-scores ranging from 0 to 100 (Mean = 50, SD = 10) for each of the seven domains. An average of all individual test T-scores was used to generate a Global T-Score (Range 0–100; Mean = 50, SD = 10), representing overall functioning. In addition to the Global and domain-level T-Scores, per standard practice, Wide Range Achievement Test (WRAT) reading scores were used to represent premorbid functioning.

#### 2.2.3. Substance Use

All participants completed a timeline follow-back (TLFB) assessment examining quantities and frequencies of all substances used across the participant’s lifetime, including METH use [41]. Variables collected included age at first use, cumulative days of use across the lifetime, and days since last use. Additionally, the computer-assisted, fully structured Composite International Diagnostic Interview (CIDI) version 2.1 [42,43] was administered to assess the presence of current and lifetime substance abuse and dependence based on DSM-IV criteria, and was used to screen out participants with any current substance use disorder (SUD) excluding METH or cannabis use disorders (CUD) [44].

### 2.3. Statistical Analysis

Two levels of statistical analyses were completed to address the present study aims. Importantly, because this was a cross-sectional study in which each participant was assessed at a single point that varied in its distance from the participant’s last METH use, all analyses model neurocognitive T-scores as a function of between-person differences in METH abstinence days and should be interpreted accordingly.

First, to examine neurocognitive functioning in association with METH abstinence days, three regression models were tested for best fit, reflecting predominant theories of recovery, with METH abstinence days as the primary predictor and Global T-Score as the outcome. A traditional linear model was tested to examine whether associations between T-scores and days of sustained METH abstinence over a two-year period were best represented by a constant positive slope. A piecewise model tested a segmented improvement in Global T-Score, with the first segment defined from 0 to 6 months, reflecting the recovery literature and clinical best practice for other substances such as alcohol, and the second segment from 6 to 24 months [24]. Finally, a linear model with a natural log-transformed METH abstinence days variable was tested to examine if associations between Global T-Score and abstinence days share a curvilinear relationship, with the most rapid improvement in early abstinence and gradually slowing over time. The best-fitting model was evaluated using the F-test for overall model fit, adjusted *R*^2,^ where higher adjusted *R*^2^ indicated greater variance explained by the predictors and thus better model fit, as well as Akaike Information Criterion (AIC) and Bayesian Information Criterion (BIC), with both lower AIC and BIC indicating better model fit [45,46,47].

Second, to examine the moderating effects of age, the Age Group variable was dummy coded and included in interaction with METH Abstinence Days to examine whether trajectories vary between the different age groups. This analysis was first completed with Global T-Score as the outcome, then repeated with each domain T-Score (i.e., Verbal Fluency T-Score, Executive Functioning T-Score, Information Processing T-Score, Learning T-Score, Recall/Memory T-Score, Attention/Working Memory T-Score, Motor Skills T-Score) to identify any domains particularly sensitive to the interaction of Age and METH abstinence. For each model, overall model fit was determined with omnibus F-tests and review of adjusted *R*^2^ values. For significant models, parameter estimates were reviewed for significance through T-Tests.

Additionally, post hoc analyses examined whether the identified effects were robust enough to be sustained with the inclusion of covariates. Candidate covariates were selected based on demonstration of significant bivariate relationships between key demographic, substance use, medical, and HIV disease characteristics and Global T-Score. Models were initially conducted including all eligible covariates, with subsequent models removing non-significant covariates. All analyses were two-tailed with an alpha level of 0.05 or confidence interval of 95% used to determine significance. For the purposes of these post hoc analyses, each individual model was considered its own family and error correction was not applied. All analyses were conducted using R in RStudio (Version 2026.04.0+526), ggplot2 [48], jtools (Version 2.3.1) [49], and boot [50].

## 3. Results

### 3.1. Sample Characteristics

The final analytic sample was composed of 122 PWH, most of whom were male (95.1%), and identified as white non-Hispanic (54.9%), Hispanic (23.0%), or black non-Hispanic (18.0%). The age of the sample ranged from 24 to 71, with an average of 44.3 (SD = 7.9) years, and 13 (SD = 2.4) years of education. Current (30-Day) cannabis use disorder (CUD) was uncommon in this group (6.1% Current CUD). On HIV Disease Characteristics, the average duration of infection was 11 years (SD = 8.0), with just over half (55.7%) reporting a historic AIDS diagnosis. Median nadir CD4 was 180 (IQR = 17, 367), while current median CD4 count was 475 (IQR = 286, 644). Most participants (75.2%) were on ART representative of prescribing practices at the time of their study visit, with nearly half (48.4%) taking some combination of Non-Nucleoside Reverse Transcriptase Inhibitors (NNRTI; e.g., Efavirenz, Nevirapine) and Protease Inhibitors (PI; e.g., Atazanavir, Ritonavir) and a quarter (24.2%) on a combination of NNRTI and Nucleoside Reverse Transcriptase Inhibitors (NRTI; e.g., Abacavir, Tenofovir). Across the total sample, 53.1% were considered virally suppressed (plasma viral load < 50 cp/mL). Mean WRAT-3/4 reading scores, representing premorbid neurocognitive ability, were in the average range (98.8, SD = 14.0). Neurocognitive T-Scores, similarly, were in the average range, with a Global T-Score average of 45.9 (SD = 6.3) and domain T-Scores ranging from 43.1 to 49.0. Examining METH use characteristics, per study inclusion criteria, all participants had a lifetime diagnosis of MUD and reported use of METH within two years (<731 days) of their study visit. Less than a quarter of participants, 16.7%, reported a current (30-day) MUD diagnosis. Participants reported an average age of first METH use of 25.9 (SD = 10.1) and a median cumulative number of days of METH use across the lifetime of 931 (IQR = 220, 3155). Participants further reported an average of 189.3 days of abstinence from METH (SD = 221.1).

See Table 1 for sociodemographic, HIV disease, cannabis use, and NC characteristics by age group, as well as significant differences identified between groups.

**Figure 1:**
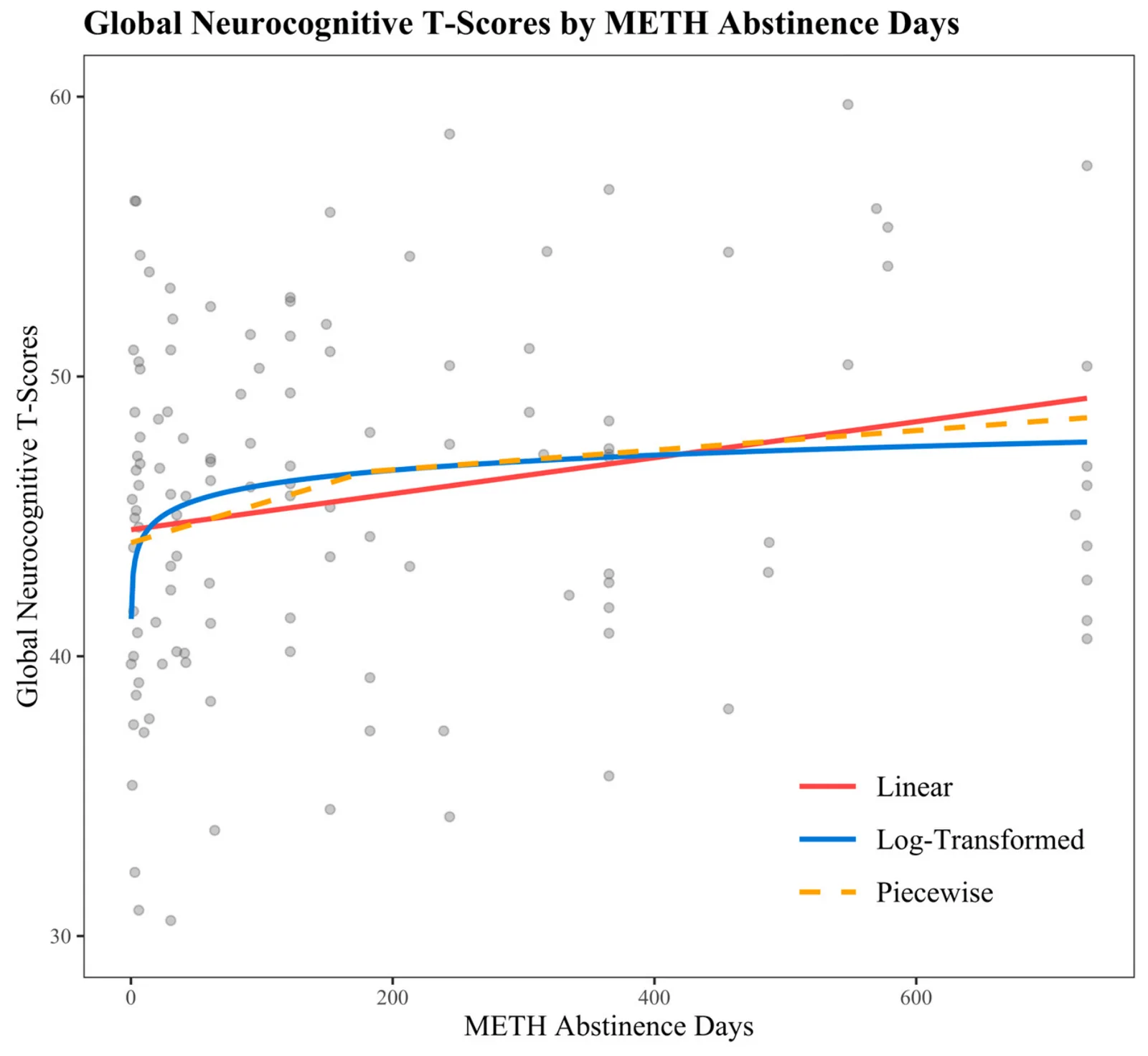
Visualization of three models fitted to represent the association of METH abstinence days with global T-scores.

Three models were fit to the Global T-score with days of METH abstinence as the predictor: a linear model, a piecewise model, and a curvilinear model with a log-transformed METH Abstinence Days variable (see Figure 1). Overall model fit was statistically significant per F-tests across the linear model (F [1, 122] = 6.04, *p* = 0.02), piecewise model (F [2, 1221] = 3.34, *p* = 0.04), and log-transformed curvilinear model (F [1, 122] = 6.52, *p* = 0.01), indicating that in each of these models the inclusion of METH Abstinence Days explained a significant portion of the variance in Global T-Score. The log-transformed model provided the best fit, though by a small margin, across all three comparative fit criteria with lowest AIC value at 815.49 as compared to the linear model at 815.95 and piecewise model at 817.29, lowest BIC at 823.95 compared to 824.42 for the linear model and 828.57 for the piecewise model, and the highest proportion of variance explained at 4.3% as compared to 3.9% for the linear model and 3.7% for piecewise model. Thus, the curvilinear model with log-transformed METH Abstinence Days variable was used across subsequent analyses.

Examining the parameters of the curvilinear model more closely revealed that log-transformed METH Abstinence Days were significantly and positively associated with Global T-Score (Estimate = 0.78, t(122) = 2.56, *p* = 0.01, 95% CI: [0.17, 1.38]) such that at higher days of METH Abstinence, Global T-Scores are also estimated to be higher. As METH abstinence days were log-transformed, the parameter estimate describes a decelerating association where differences in estimated Global T-Score were largest across early METH abstinence days and grew smaller with more METH abstinence days.

### 3.2. Interactions with Age Group

To test whether the association between METH Abstinence Days and neurocognitive test T-Scores varied by age, a dummy-coded interaction term between METH Abstinence Days and Age Group (i.e., <42 [Reference Group], 42–48, 49+) was included in the analysis. Initial models only included variables of primary interest (i.e., no covariates).

For the Global T-score, the overall model fit was significant, F(5, 118) = 2.63, *p* = 0.03 (adjusted *R*^2^ = 0.062). Looking at individual parameters, only the main effect of log-transformed METH Abstinence Days demonstrated significant contributions to the model (Estimate = 1.2, *t*(118) = 2.26, *p* = 0.03, 95% CI: [0.14, 2.16]). The main effect of Age Group and interaction effects between Age Group and METH Abstinence Days were not significant.

At the domain level, only two models demonstrated overall model fit: Information Processing Speed and Working Memory (see Table 2). The remaining models were not significant in the omnibus test of fit, including Verbal Fluency, Executive Functioning, Learning, Recall, and Motor Skills, and thus were not further interpreted. 

**Figure 2:**
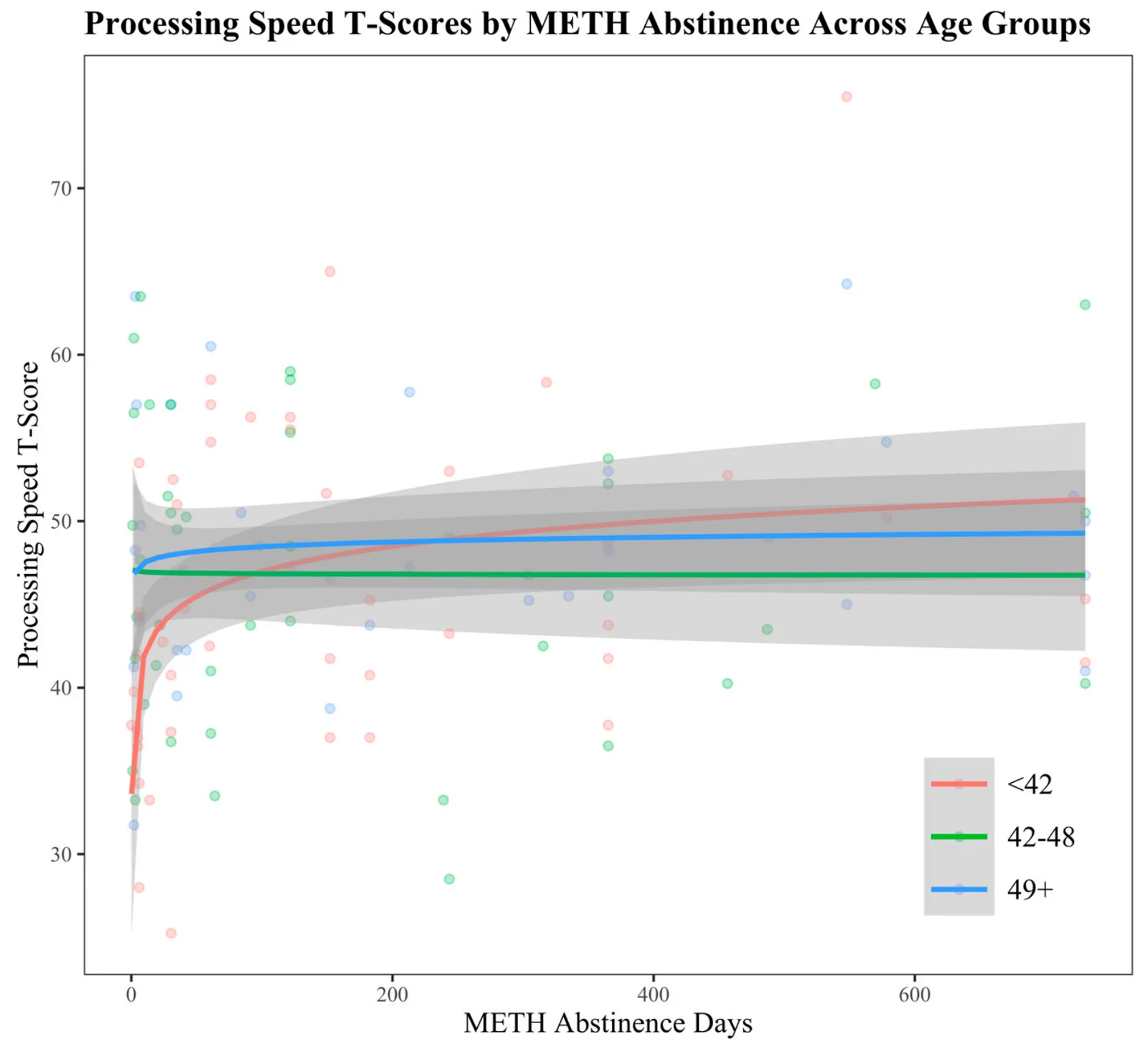
Interaction between Age Group and METH Abstinence Days in association with outcome.

Information Processing Speed T-Scores: For Information Processing Speed, a significant main effect of METH Abstinence Days was identified (Estimate = 2.17, *p* < 0.01). A marginally significant interaction effect between METH Abstinence Days and Age Group was also identified for Information Processing Speed (*F*(2, 118) = 2.93, *p* = 0.057). This overall interaction was driven by a significant interaction effect between METH Abstinence Days and the 42–48 Age Group (Estimate = −2.21, *p* = 0.02) alone. Though no overall main effect was found for Age Group (*F*(2, 120) = 0.78, *p* = 0.46), simple slopes revealed significant differences for the 42–48 group (Estimate = 10.06, *p* = 0.02) and marginally significant for the 49+ group (Estimate = 9.57, *p* = 0.05), suggesting differences in baseline performance from the reference <42 group (see Figure 2).

No significant interaction effects between Age Group and METH Abstinence Days were identified for the Working Memory domain. Even so, main effects of log-transformed METH Abstinence Days (Estimate = 1.3, *t* [117] = 2.07, *p* = 0.04, 95% CI: [0.06, 2.58]) and Age Group were identified (*F*(2, 119) = 6.40, *p* < 0.01). Group main effects were largely driven by significant differences between the 49+ Age Group and the reference <42 Age Group (Estimate = 12.7, *p* < 0.01). This effect reflects a baseline difference in Working Memory, independent of days of abstinence, such that the older group had better Working Memory T-Scores. It is important to recognize here that the T-Scores are adjusted for age. Higher T-Scores in older groups do not necessarily reflect better raw performance. Raw scores could be higher in the younger group, but performance relative to age group appears to be better in this older group. Parameter estimates of each significant model excluding covariates are presented in Table 3.

In the examination of robust effects, covariates were included in subsequent models and selected based on the demonstration of significant bivariate relationships between key demographic, substance use, medical, and HIV disease characteristics and Global T-Score. Education Years, WRAT 3/4 Scores, Nadir CD4 T-Cells, and METH Age of First Use demonstrated significant relationships with Global T-Score. These four variables were included in initial models; however, across all outcomes (i.e., Global T-Score and domain T-Scores), Education Years and METH Age of First Use were not significant predictors and therefore were removed. Thus, all remaining models included only WRAT 3/4 Score and Nadir CD4 T-Cells.

Inclusion of covariates yielded minimal changes in overall effects observed. For the Global T-score, the overall model was significant, F(7, 109) = 6.16, *p* < 0.01 (adjusted *R*^2^ = 0.237), as were the overall models for Executive Function (F [7, 109] = 5.08, *p* < 0.01 [adjusted *R*^2^ = 0.197]), Information Processing Speed (F [7, 109] = 5.19, *p* < 0.01 [adjusted *R*^2^ = 0.101]), Learning (F [7, 109] = 2.31, *p* < 0.01 [adjusted *R*^2^ = 0.073]), Working Memory (F [7, 109] = 5.75, *p* < 0.01 [adjusted *R*^2^ = 0.223]), and Motor (F [7, 109] = 2.87, *p* < 0.01 [adjusted *R*^2^ = 0.102]). The remaining models were not significant in the omnibus test of fit, including, again, Verbal Fluency (F [7, 108] = 1.81, *p* = 0.09) and Recall (F [7, 109] = 1.70, *p* = 0.12) and thus were not further interpreted.

As in the non-covariate models, significant interactions between METH Days of Abstinence and age were observed. Abstinence and Age Group were, again, only found in Information Processing Speed (*F*(2, 109) = 3.13, *p* = 0.048), driven by simple slope associations between METH Days of Abstinence and the 42–48 Age Group (Estimate = −2.15, *p* = 0.02). While a main effect of Age Group was, again, not identified for Information Processing Speed, simple slopes additionally reveal significant differences for both the 42–49 Age Group (Estimate = 10.1, *p* = 0.01) and the 49+ Age Group (Estimate = 9.8, *p* = 0.03). Complete results of the significant models, including covariates, are presented in Table 4.

## 4. Discussion

In this study, we examined the correspondence between length of METH abstinence and neurocognitive performance, along with the moderating role of age, in a cross-sectional sample of PWH with a history of MUD, in order to glean a likely trajectory of neurocognitive recovery. Recognizing the limitations of the cross-sectional design, three findings warrant emphasis. First, the association between METH abstinence and global neurocognitive functioning was best represented by a curvilinear rather than a linear or piecewise model, such that gains were concentrated in the earliest period of abstinence and decelerated thereafter. Second, this relationship was largely consistent across age, contrary to our hypothesis that recovery would be steeper in younger individuals. Third, age did moderate the effect of abstinence on Information Processing Speed, a cognitive domain that is especially vulnerable to aging, and persisted after adjustment for premorbid functioning and nadir CD4.

The curvilinear shape of the relationship between length of abstinence and cognition observed here aligns closely with the broader substance-recovery literature, in which improvement following sustained abstinence is time-dependent, with a large proportion of improvement in function occurring in the early weeks-to-months before it gains plateau [37]. This comes as no surprise, as this pattern is consistent with existing research across substances highlighting that neurocognitive recovery in association with abstinence is often front-loaded [24]. Nevertheless, this is among the first demonstrations of such associations explicitly in PWH who used METH, accounting for the role of age. It is important to note that the modest proportion of variance explained reinforces that abstinence-related recovery in this population is partial, consistent with evidence that certain effects of chronic METH exposure, particularly in the context of ongoing neuroHIV, may be longer-lasting and that neurocognitive function may be more strongly defined by other HIV-related disease processes rather than METH use alone [34].

Contrary to hypotheses, in this sample, Age Group did not prove to be a significant moderator of the relationship between METH Abstinence Days and Neurocognitive T-Scores. It is worth noting that our oldest group had a number of possible advantages, including greater education, a much higher proportion with undetectable viral load, a lower proportion of current MUD, and tended to have somewhat better (demographically adjusted) neurocognitive scores across the battery, possibly affecting our ability to detect Age Group effects. The only exception to this was in the Information Processing Speed domain, which also emerged as the domain most sensitive to abstinence across the overall sample (i.e., largest effect sizes). There is strong evidence that Information Processing Speed is both among the neurocognitive domains most impacted by dopaminergic dysregulation, to which both HIV and METH contribute, and has also been shown to be consistently impaired by HIV and METH independently across studies [15,51]. It follows that Information Processing Speed, as a domain likely to be impacted by METH use in the first place, has potential for neurocognitive recovery in the context of abstinence and its associated neurobiological changes (i.e., dopamine transporter availability and thalamic glucose metabolism) [18,20].

These neurobiological underpinnings may also explain the age effect specific to Information Processing Speed. As normal aging is similarly accompanied by disruptions to dopaminergic pathways and other neurodegenerative processes [52], processing-speed recovery may be diminished in older PWH due to the cumulative burden of aging and HIV in addition to METH-related neurotoxicity [53,54]. This is consistent with prior evidence that abstinence-related gains and the residual legacy effects of remote METH exposure are not as easily detectable in older PWH [34,35]. In short, as Information Processing Speed is especially impacted by aging, neurocognitive recovery in the context of METH abstinence may be limited by aging with HIV [27,55,56].

In considering the implications of these findings, it is also important to recognize changes in METH use rates and practices as individuals age and the increasing reports of older adults in treatment or critical care due to METH use [7]. In the present study, our oldest group (>49) had an average age of 53.7, reflecting more of a middle-aged sample rather than a proper older-adult sample [57]. As much of the existing literature on METH use and its impacts focuses on younger and middle-aged adults, it remains an open and important question whether the associations between abstinence and neurocognitive recovery observed in other studies generalize to truly older PWH with MUD. Larger effect sizes and impacts across broader domains may become more apparent in future studies that are able to target more of this aging population.

## 5. Limitations and Strengths

Several limitations should be considered in interpreting these findings. First and foremost, this was a cross-sectional study in which each participant was assessed at a single point that varied in its distance from the last METH use. All trajectories therefore modeled between-person differences in abstinence duration rather than within-person change over time. This design leaves open the possibility of reverse-directional effects, where individuals with better-preserved neurocognitive functioning are more able to achieve and sustain abstinence, rather than abstinence driving the observed differences. Furthermore, the present study period spanned two years, largely restricted by parent study inclusion criteria. This design choice does not capture the continued improvement that may accrue beyond the first two years. Though the present study findings and past research provide evidence that the steepest associations between abstinence and neurocognition are in the early weeks of abstinence, future research may consider longer windows of time to study neurocognitive associations with longer-term abstinence [24,37]. A further limitation concerns how recovery itself was conceptualized. The present study modeled neurocognitive functioning as a function of days of sustained abstinence and therefore speaks specifically to abstinence-based recovery. The present study does not account for neurocognitive gains that may be achieved through non-abstinent pathways, such as reductions in the frequency or quantity of METH use that fall short of abstinence. Given growing recognition that meaningful clinical and functional benefit can follow reduced-use and harm-reduction trajectories rather than abstinence alone, the recovery estimated here is best understood as reflecting one route to neurocognitive improvement rather than the only one. Whether, and to what degree, partial reductions in use are associated with neurocognitive benefit and how any such benefit compares to that observed with sustained abstinence remains an important open question. An important consideration is that the mean global and domain-level performance fell largely within normal limits, with only 17.2% classified as neurocognitively impaired based on a Global T-score < −1 standard deviation, constraining the magnitude of neurocognitive improvement that can be detected. To the extent that individuals with greater baseline impairment have the most room to improve, the present estimates may understate the effects of sustained abstinence achievable among more impaired participants, and the trajectories observed here may not generalize to samples enriched for neurocognitive impairment. Additionally, the sample was modest in size and relatively homogeneous demographically, limiting generalizability beyond this sample. Finally, while the present study excluded participants with a current substance use disorder other than MUD and CUD to isolate the effects of METH specifically, this approach did not exclude or control for subclinical levels (i.e., use not meeting use disorder criteria) of other substance use. Existing research on the neurocognitive impact of subclinical substance use remains limited and has yielded mixed findings across substances, with much research suggesting little impact of subclinical levels of use [58,59,60]. Nevertheless, the possibility that co-occurring subclinical use could confound associations between METH abstinence and neurocognitive functioning remains.

These limitations are balanced by notable strengths. The study drew on data across a comprehensive validated neurocognitive battery yielding demographically adjusted domain- and global-level T-scores, with medical and HIV-disease characterization as well as standardized substance use histories. Furthermore, rather than assuming a recovery shape, we formally compared competing linear, step, and curvilinear trajectories against one another using multiple fit criteria, and translated the best-fitting model into clinically interpretable, bootstrapped estimates of when recovery milestones are reached. Finally, by focusing on PWH specifically, the study addresses a gap in the literature by explicitly and exclusively addressing this population.

## 6. Conclusions

In a sample of PWH with a history of MUD, higher neurocognitive scores were associated with greater METH Abstinence in a curvilinear model, with higher values concentrated early in abstinence and decelerating over time. On the domain-level, Information Processing Speed emerged as both the most abstinence-sensitive domain and the only one in which the relationship between neurocognition and abstinence was moderated by age. These findings should be regarded as hypothesis-generating and, given the cross-sectional design, this research would be best followed up with confirmation in longitudinal studies that track neurocognitive change within individuals, extend follow-up beyond two years to characterize the true asymptote of recovery, and are adequately powered to test age moderation at the domain level. Furthermore, while population-level rates of METH use are generally lower among older adults relative to younger and middle-aged adults, rates of METH use and treatment-seeking in older populations have been rising in recent years, highlighting the need to understand potential unique trajectories of neurocognitive recovery specifically in older adults [1,31]. These findings have important implications not only for research but future clinical practice as well. The front-loaded nature of recovery suggests that the earliest weeks-to-months of abstinence are a period of meaningful neurocognitive change and a high-value window for support. Neurocognitively demanding assessments and interventions, such as cognitive rehabilitation, skills-based relapse-prevention work, or psychoeducation with substantial learning demands, are likely to be more effective after substantive abstinence has been achieved. The prominence of Information Processing Speed points to a potential target for monitoring and intervention, and the age-related attenuation of its recovery underscores the importance of setting realistic, age-informed expectations, particularly for older PWH. Importantly, evidence for measurable neurocognitive benefit accompanying sustained abstinence offers a concrete, motivating message for prevention and treatment efforts in this population.

## Figures and Tables

**Table 1:** Participant demographics and clinical characteristics (*N* = 124).

	Age Group			
Demographics	<42 ^A^ *(n = 45)*	42–48 ^B^ *(n = 44)*	49+ ^C^ *(n = 33)*	Significant Differences
Age (years)	36.6 (5.3)	45.2 (1.5)	53.7 (4.4)	-
Education Years	12.9 (2.3)	12 (2.2)	13.6 (2.7)	-
Sex (% male)	97.8%	93.2%	93.9%	-
Race/Ethnicity				
* % White non-Hispanic*	44.4%	52.3%	72.7%	C > A *
* % Black non-Hispanic*	20.0%	13.6%	21.2%	-
* % Hispanic/Latino*	26.7%	31.8%	6.1%	A *, B ** > C
* % Asian*	4.4%	0.0%	0.0%	-
* % Other*	4.4%	2.3%	0.0%	-
HIV Disease Characteristics				
AIDS Diagnosis (% AIDS)	53.3%	59.1%	54.6%	
Est. Duration of HIV (years)	8.6 (6.9)	10.9 (7.6)	14.4 (8.9)	C > A **
CD4+ T-cells (cells/uL)	392 [158.3, 619.5]	483 [306.5, 790]	503 [336.3, 659]	-
Nadir CD4+ T-cells (cells/uL)	170.5 [11.3, 333]	139 [12, 395]	198.5 [77, 322.5]	-
ART (% on ART)	75.0%	75.0%	75.8%	-
* % on NRTI + PI*	48.5%	54.6%	40.0%	
* % on NRTI + NNRTI*	30.3%	21.2%	20.0%	
* % on NRTI + II*	3.0%	15.2%	12.0%	
* % on 3+ Class Combination*	18.2%	9.1%	28.0%	
Viral Suppression on ART (% Undetectable)	59.3%	64.5%	92.0%	C > B *, A **
Neurocognitive				
WRAT-3/4 Score	98.1 (13.4)	98.7 (15.2)	99.8 (13.6)	-
Global T-Score	45.1 (7.0)	45.0 (6.1)	48.1 (5.3)	-
Verbal T-Score	49.0 (8.7)	47.3 (8.1)	51.1 (8.7)	-
Executive Function T-Score	44.3 (9.9)	45.7 (7.8)	49.5 (7.0)	C > A **
Processing Speed T-Score	45.7 (9.7)	46.9 (8.8)	48.0 (6.9)	-
Learning T-Score	42.8 (11.0)	41.7 (8.2)	45.5 (10.3)	-
Recall T-Score	43.1 (9.7)	43.0 (8.2)	45.9 (8.7)	-
Working Memory T-Score	43.9 (8.3)	44.0 (7.9)	49.6 (7.5)	C > B **, A ***
Motor T-Score	42.7 (9.7)	43.6 (12.1)	44.7 (11.8)	-
Substance Use History				
Current MUD (% Diagnosed)	25.6%	13.6%	9.7%	-
METH Age of First Use	23.1 (7.9)	24.5 (7.9)	31.9 (12.7)	C > B **, A ***
METH Abstinence Days	157.2 (195.6)	177.5 (215.8)	248.6 (253.8)	-
METH Lifetime Days	1513 (1918.9)	2434 (2397.1)	2175.1 (2567.9)	-
Current CUD (% Diagnosed)	5.1%	4.6%	9.7%	-

Note: Mean and standard deviation are indicated as Mean (standard deviation). Median and interquartile range are indicated as Median [IQR 25th Percentile, IQR 75th Percentile]. Percentages indicate column percentages. Italics indicate characteristics nested within a larger category. NRTI = Nucleoside Reverse Transcriptase Inhibitors, NNRTI = Non-Nucleoside Reverse Transcriptase Inhibitors, PI = Protease Inhibitor, II = Integrase Inhibitor. A = <42 Group, B = 42–48 Group, C = 49+ Group. * *p* < 0.05 ** *p* < 0.01 *** *p* < 0.001.

**Table 2:** Overall model fit for all log-transformed models.

Outcome	DF	*F*-Ratio	*p*-Value	*R* ^2^	AIC	BIC
Global	118	2.63	0.03 *	0.06	816.83	836.57
Verbal Fluency	117	0.94	0.46	0.00	884.77	904.46
Executive Functioning	118	2.07	0.07	0.04	888.94	908.68
Information Processing Speed	118	2.53	0.03 *	0.06	888.94	908.69
Learning/Memory	117	1.06	0.39	0.00	926.30	945.98
Recall	117	1.24	0.30	0.01	905.06	924.74
Working Memory	117	3.98	<0.01 *	0.11	863.43	883.11
Motor Speed	117	1.99	0.08	0.04	947.35	967.04

Note: DF = Degrees of Freedom; AIC = Akaike Information Criterion; BIC = Bayesian Information Criterion. * *p* < 0.05.

**Table 3:** Regression results for all models.

Global T-Score	Estimate	SE	T-Ratio	*p*-Value	95% CI
Intercept	40.13	2.22	18.11	<0.001 ***	35.74, 44.52
METH Abstinence Days Log	1.15	0.51	2.26	0.026 *	0.14, 2.16
Age: 42–48	2.52	3.13	0.8	0.42	−3.69, 8.72
Age: 49+	6.74	3.54	1.9	0.06	−0.28, 13.75
METH Abstinence Days Log × Age: 42–48	−0.55	0.71	−0.78	0.44	−1.97, 0.86
METH Abstinence Days Log × Age: 49	−0.88	0.77	−1.15	0.25	−2.4, 0.63
Information Processing Speed					
Intercept	37	2.96	12.48	<0.001 ***	31.13, 42.87
METH Abstinence Days Log	2.17	0.68	3.18	<0.01 **	0.82, 3.52
Age: 42–48	10.06	4.19	2.4	0.02 *	1.76, 18.35
Age: 49+	9.57	4.74	2.02	0.05	0.19, 18.95
METH Abstinence Days Log × Age: 42–48	−2.21	0.95	−2.32	0.02 *	−4.1, −0.33
METH Abstinence Days Log × Age: 49	−1.76	1.02	−1.72	0.09	−3.79, 0.27
Working Memory					
Intercept	38.71	2.75	14.05	<0.001 ***	33.25, 44.16
METH Abstinence Days Log	1.32	0.64	2.07	0.04 *	0.06, 2.58
Age: 42–48	2.87	3.89	0.74	0.46	−4.83, 10.57
Age: 49+	12.73	4.4	2.89	<0.01 **	4.02, 21.43
METH Abstinence Days Log × Age: 42–48	−0.72	0.89	−0.81	0.42	−2.48, 1.04
METH Abstinence Days Log × Age: 49	−1.66	0.95	−1.74	0.09	−3.55, 0.23

Note: SE = standard error; 95% CI = 95% confidence interval. * *p* < 0.05 ** *p* < 0.01 *** *p* < 0.001.

**Table 4:** Regression results for all models including covariates.

Global T-Score	Estimate	SE	T-Ratio	*p*-Value	*95% CI*
Intercept	24.54	4.28	5.73	<0.001	16.05, 33.03
METH Abstinence Days Log	1.11	0.48	2.29	0.02 *	0.15, 2.07
Age: 42–48	2.33	2.95	0.79	0.43	−3.51, 8.18
Age: 49+	7.37	3.25	2.27	0.03 *	0.92, 13.82
Nadir CD4	0.01	0	2.46	0.02 *	0, 0.01
WRAT Score	0.14	0.04	3.68	<0.001	0.07, 0.22
METH Abstinence Days Log × Age: 42–48	−0.43	0.68	−0.63	0.53	−1.78, 0.92
METH Abstinence Days Log × Age: 49	−1.04	0.71	−1.47	0.15	−2.44, 0.36
Executive Function					
Intercept	16.98	5.92	2.87	<0.001	5.25, 28.72
METH Abstinence Days Log	0.77	0.67	1.14	0.26	−0.56, 2.09
Age: 42–48	2.21	4.08	0.54	0.59	−5.88, 10.29
Age: 49+	10.1	4.5	2.25	0.03 *	1.19, 19.02
Nadir CD4	0	0	0.57	0.57	−0.01, 0.01
WRAT Score	0.24	0.05	4.46	<0.001	0.13, 0.35
METH Abstinence Days Log × Age: 42–48	−0.13	0.94	−0.14	0.89	−1.99, 1.73
METH Abstinence Days Log × Age: 49	−1.25	0.98	−1.28	0.2	−3.18, 0.69
Information Processing Speed					
Intercept	16.82	5.88	2.86	<0.01 **	5.17, 28.48
METH Abstinence Days Log	2.07	0.67	3.11	<0.01 **	0.75, 3.39
Age: 42–48	10.09	4.05	2.49	0.01 *	2.06, 18.12
Age: 49+	9.85	4.47	2.2	0.03 *	0.99, 18.7
Nadir CD4	0.01	0	2.06	0.04 *	0, 0.01
WRAT Score	0.19	0.05	3.61	<0.001 ***	0.09, 0.3
METH Abstinence Days Log × Age: 42–48	−2.15	0.93	−2.31	0.02 *	−4, −0.31
METH Abstinence Days Log × Age: 49	−1.91	0.97	−1.96	0.05	−3.83, 0.02
Learning					
Intercept	25	7.46	3.35	<0.001 ***	10.22, 39.78
METH Abstinence Days Log	1.45	0.84	1.71	0.09	−0.23, 3.12
Age: 42–48	1.31	5.14	0.25	0.80	−8.87, 11.49
Age: 49+	8.89	5.67	1.57	0.12	−2.34, 20.12
Nadir CD4	0.01	0	2.15	0.03 *	0, 0.02
WRAT Score	0.09	0.07	1.39	0.17	−0.04, 0.23
METH Abstinence Days Log × Age: 42–48	−0.61	1.18	−0.52	0.61	−2.96, 1.73
METH Abstinence Days Log × Age: 49	−1.54	1.23	−1.25	0.21	−3.98, 0.9
Working Memory					
Intercept	24.4	5.38	4.53	<0.001	13.73, 35.07
METH Abstinence Days Log	1.02	0.61	1.68	0.10	−0.18, 2.23
Age: 42–48	0.21	3.71	0.06	0.95	−7.14, 7.56
Age: 49+	12.01	4.09	2.94	<0.01 **	3.91, 20.12
Nadir CD4	0.01	0	2.3	0.02 *	0, 0.01
WRAT Score	0.14	0.05	2.91	<0.01 **	0.05, 0.24
METH Abstinence Days Log × Age: 42–48	−0.04	0.85	−0.05	0.96	−1.74, 1.65
METH Abstinence Days Log × Age: 49	−1.58	0.89	−1.78	0.08	−3.34, 0.18
Motor					
Intercept	25.93	8.16	3.18	<0.001	9.76, 42.09
METH Abstinence Days Log	2.34	0.92	2.53	0.01 *	0.51, 4.17
Age: 42–48	5.97	5.62	1.06	0.29	−5.16, 17.11
Age: 49+	5.49	6.2	0.89	0.38	−6.79, 17.77
Nadir CD4	0.01	0.01	2.54	0.01 *	0, 0.02
WRAT Score	0.05	0.07	0.65	0.52	−0.1, 0.2
METH Abstinence Days Log × Age: 42–48	−1.29	1.29	−1	0.32	−3.86, 1.27
METH Abstinence Days Log × Age: 49	−1.07	1.35	−0.8	0.43	−3.74, 1.59

Note: SE = standard error; 95% CI = 95% confidence interval. * *p* < 0.05 ** *p* < 0.01 *** *p* < 0.001.

## Data Availability

Data are maintained in secure repositories at the HIV Neurobehavioral Research Program, University of California San Diego, and are available upon request via email at hnrpresource@ucsd.edu.

## Abbreviations

The following abbreviations are used in this manuscript.

AIC	Akaike Information Criterion
ART	Antiretroviral Therapy
BIC	Bayesian Information Criterion
CIDI	Composite International Diagnostic Interview
HNRP	HIV Neurobehavioral Research Program
LLQ	Lower limit of quantitation
PWH	People with HIV
MAT	Medication for addiction treatment
METH	Methamphetamine
MUD	Methamphetamine Use Disorder
NCI	Neurocognitive Impairment
SUD	Substance Use Disorder
TLFB	Timeline follow-back
WRAT	Wide Range Achievement Test
